# In Vitro Activity of Novel β-Lactam/β-Lactamase Inhibitors Against Carbapenem-Resistant *Pseudomonas aeruginosa* and *Enterobacterales* in Korea

**DOI:** 10.3390/antibiotics14070649

**Published:** 2025-06-26

**Authors:** Seulgi Moon, Jongyoun Yi, Mee Kyung Ko, Yong Ki Sim, Kye-Hyung Kim

**Affiliations:** 1 Department of Laboratory Medicine, Pusan National University School of Medicine, Busan 49241, Republic of Korea; sulggimoon@pusan.ac.kr (S.M.); socioliberal@pusan.ac.kr (J.Y.); 2 Biomedical Research Institute, Pusan National University Hospital, Busan 49241, Republic of Korea; qeqazwsx@pnuh.co.kr; 3 Department of Internal Medicine, Pusan National University School of Medicine, Busan 49241, Republic of Korea; asla11@naver.com

**Keywords:** antimicrobial agents, carbapenem resistance, bacteremia, β-lactamase inhibitors, Korea, susceptibility

## Abstract

**Background/Objectives:** Carbapenem-resistant *Enterobacterales* (CRE) and carbapenem-resistant *Pseudomonas aeruginosa* (CRPA) are challenging multidrug-resistant pathogens. This study evaluated the in vitro susceptibility of CRE and CRPA blood isolates from Korea to novel β-lactam/β-lactamase inhibitor combinations: ceftolozane/tazobactam (C/T), ceftazidime/avibactam (CZA), imipenem/cilastatin/relebactam (IMR), and meropenem/vaborbactam (MEV). **Methods:** Blood isolates of CRE (*n* = 55) and CRPA (*n* = 65) collected between September 2017 and September 2022 in a Korean tertiary hospital were included. Carbapenemase production was determined using phenotypic and molecular methods. In vitro susceptibility to C/T, CZA, IMR, and MEV was determined primarily by broth microdilution using current CLSI/EUCAST breakpoints. Clinical characteristics and in-hospital mortality were retrospectively reviewed. **Results:** Among non-carbapenemase-producing (non-CP) CRPA isolates (*n* = 47), susceptibility rates were 83.0% to C/T and 70.2% to CZA. For KPC-producing CRE isolates (*n* = 28), susceptibility rates were high to CZA (92.9%), IMR (82.1%), and MEV (96.4%). However, non-CP CRE isolates (*n* = 22) showed low susceptibility to C/T (18.2%) but high susceptibility to CZA (100%), IMR (81.8%), and MEV (95.5%). CRE infections were associated with higher rates of hematologic malignancy, immunosuppression, and in-hospital mortality (63.6% vs. 18.5% for CRPA, *p* < 0.001). **Conclusions:** The susceptibility of CRE and CRPA to novel β-lactam/β-lactamase inhibitors varies significantly by species and carbapenemase production. CZA, IMR, and MEV showed promising activity against KPC-producing CRE. These findings can inform empirical therapy and stewardship efforts in Korea.

## 1. Introduction

The rising prevalence of antimicrobial resistance represents a critical global health challenge. The rapid spread of antibiotic-resistant pathogens has led to increased mortality, prolonged hospital stays, and elevated healthcare costs in patients with infectious diseases [1]. The emergence of multidrug-resistant (MDR) bacteria, especially Gram-negative organisms that have demonstrated resistance to multiple antibiotic classes and continue to disseminate globally, is particularly concerning [2].

Among Gram-negative bacteria, carbapenem resistance has been increasingly reported in hospital-acquired pathogens such as *Pseudomonas aeruginosa* and *Acinetobacter baumannii*, as well as community-associated *Escherichia coli*, *Klebsiella pneumoniae*, and *Enterobacter cloacae* isolates [3]. These carbapenem-resistant organisms (CROs) are associated with limited treatment options, increased healthcare burden, and poor clinical outcomes.

Carbapenem-resistant *Enterobacterales* (CRE) are commonly classified as either carbapenemase-producing (CP-CRE) or non-carbapenemase-producing (non-CP CRE) [2,4,5]. In non-CP CRE, resistance typically arises from decreased outer membrane permeability—such as porin loss—combined with the presence of extended-spectrum β-lactamases (ESBLs) or AmpC enzymes, which do not efficiently hydrolyze carbapenems but contribute to resistance in synergy with permeability defects [6,7]. Ambler’s classification categorizes carbapenemases into class A (e.g., *bla*_KPC_, *bla*_GES_), B metallo-β-lactamases (e.g., *bla*_NDM_, *bla*_IMP_, *bla*_VIM_), and D enzymes (e.g., *bla*_OXA-48-like_) [8]. Each enzyme type exhibits species-specific distributions, with *bla*_KPC_ being predominant in *K. pneumoniae* and *bla*_NDM_ increasingly identified in both *Enterobacterales* and *P. aeruginosa*. In *P. aeruginosa*, carbapenem resistance may arise through multiple mechanisms, including decreased expression of outer membrane porins, overexpression of efflux pumps, and carbapenemase production. Carbapenemase-producing *P. aeruginosa* (CP-CRPA) strains often harbor genes such as *bla*_KPC_, *bla*_NDM_, *bla*_VIM_, *bla*_IMP_, and *bla*_OXA-48-like_, which facilitate the rapid dissemination of resistance [9].

The incidence of CRE and CRPA has continued to rise globally [5,10]. The coronavirus disease (COVID-19) pandemic has further exacerbated this trend due to increased antibiotic usage, disruptions in infection control, and laboratory testing limitations. In the United States, hospital-acquired CRE and MDR *P. aeruginosa* infections rose by 35% and 32%, respectively, in 2020 compared to those in 2019 [10]. In Korea, antimicrobial consumption increased by 3.4% to 25.9% in multiple hospitals during the post-COVID-19 period, accompanied by increases of 22.4% and 20.1% in CRE and CRPA prevalences, respectively [11]. Furthermore, national surveillance data (Kor-GLASS) have indicated that overall antibiotic consumption, particularly of broad-spectrum cephalosporins, increased in the years following 2021 compared to the pre-pandemic period (2014–2019). Concurrently, resistance rates in key pathogens like *Escherichia coli* and *Klebsiella pneumoniae* to several antibiotics also showed non-decreasing or even increasing trends up to 2023, highlighting the ongoing and complex challenge of antimicrobial resistance in the post-COVID-19 era in Korea [12]. Regional variation in the epidemiology of carbapenemase-producing organisms has also been observed in Korea, likely reflecting differences in antimicrobial usage patterns and infection control practices.

Recently, novel β-lactam/β-lactamase inhibitor combinations—including ceftolozane/tazobactam (C/T), ceftazidime/avibactam (CZA), imipenem/cilastatin/relebactam (IMR), and meropenem/vaborbactam (MEV)—have been introduced as promising agents for treating CRE and CRPA infections [13]. These agents have only recently become available in Korea, and local data regarding their in vitro activity and clinical efficacy remain scarce, particularly for isolates collected in recent years [14].

This study aimed to address the scarcity of Korean data on the in vitro activity and clinical efficacy of these novel agents, especially against recently collected CRE and CRPA isolates, by investigating the clinical characteristics and antimicrobial susceptibility profiles of CRE and CRPA bloodstream isolates from a Korean tertiary hospital. Specifically, these blood isolates were evaluated for the distribution of carbapenemase genes, and their in vitro activity of C/T, CZA, IMR, and MEV was assessed. Our findings are expected to contribute valuable insights into the potential clinical role of these novel agents in the Korean healthcare setting.

## 2. Results

### 2.1. Patient Characteristics

**Figure 1:**
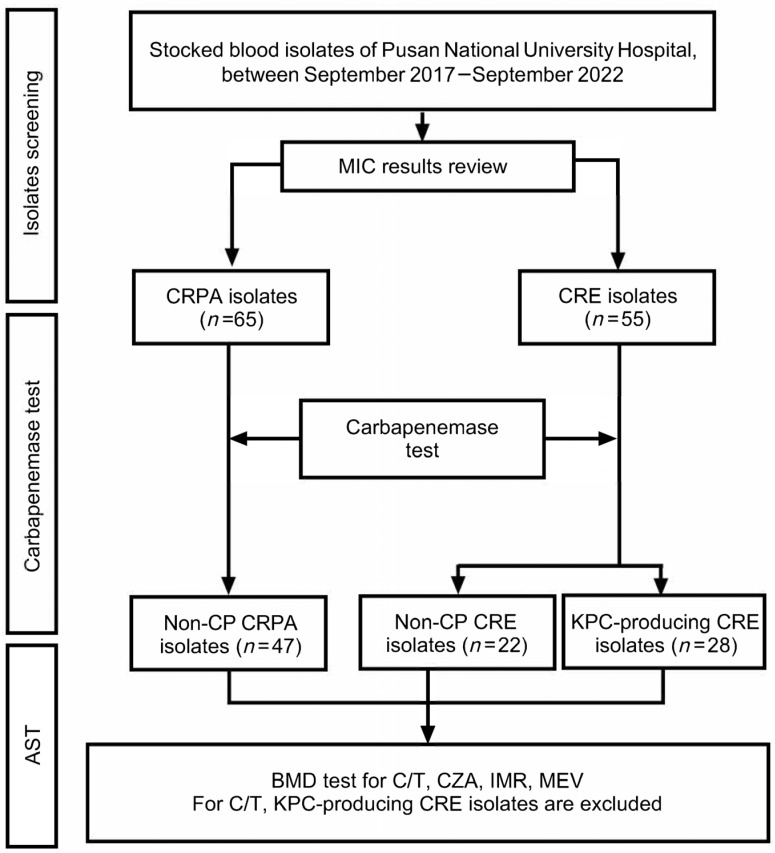
Flowchart of isolate selection and classification for antimicrobial susceptibility testing. Blood isolates of *Pseudomonas aeruginosa* and *Enterobacterales* collected at Pusan National University Hospital between September 2017 and September 2022 were screened for carbapenem resistance. Following MIC review, CRPA and CRE isolates underwent carbapenemase testing. Based on the results, isolates were categorized as non-CP CRPA, non-CP CRE, or KPC-producing CRE. Notably, KPC-producing CRE isolates were excluded from C/T susceptibility testing. Abbreviations used are defined in the list at the end of the manuscript.

A total of 120 bloodstream isolates—65 CRPA and 55 CRE—were included. Antimicrobial susceptibility testing for C/T, CZA, IMR, and MEV was performed on 97 isolates (Figure 1). Patients in the CRPA group were significantly older than those in the CRE group (median age, 65 vs. 55 years; *p* = 0.008) (Table 1). Sex distribution was similar (60% male in both groups; *p* = 0.451). Underlying comorbidities differed notably between the two groups: the CRE group had higher rates of chronic lung disease or COPD (7.3% vs. 0%; *p* = 0.041), hematologic malignancy (25.5% vs. 4.6%; *p* < 0.001), and immunosuppressive therapy (60.0% vs. 15.4%; *p* < 0.001), indicating a higher baseline risk profile. The primary source of bacteremia differed significantly (Table 2). Urinary tract infection was more frequent among CRPA patients (24.6% vs. 1.8%; *p* < 0.001), whereas primary bacteremia or neutropenic fever predominated among CRE patients (23.6% vs. 6.2%; *p* = 0.008). The use of invasive devices at infection onset—including central venous catheters (70.9% vs. 35.4%; *p* < 0.001), mechanical ventilation (29.1% vs. 6.2%; *p* = 0.001), and nasogastric tubes (38.2% vs. 20.0%; *p* = 0.041)—was significantly more common in the CRE group. Differences in empirical antibiotic use were also observed (Table 3). Combination therapy with colistin and carbapenem was more frequent in CRE-infected patients (18.2% vs. 1.5%; *p* = 0.003), while cephalosporin monotherapy was more common among CRPA-infected patients (26.2% vs. 10.9%; *p* = 0.039). Differences in empirical antibiotic use were also observed (Table 3). Combination therapy with colistin and carbapenem was more frequent in CRE-infected patients (18.2% vs. 1.5%; *p* = 0.003), while cephalosporin monotherapy was more common among CRPA-infected patients (26.2% vs. 10.9%; *p* = 0.039). Notably, a substantial portion of patients in both groups (30.8% of CRPA and 38.2% of CRE) did not receive targeted therapy for the identified resistant pathogen. In-hospital mortality was significantly higher in the CRE group (63.6%) compared to the CRPA group (18.5%; *p* < 0.001).

### 2.2. Distribution of Carbapenemases

Carbapenemase production and the presence of the five major carbapenemase genes (KPC, NDM, VIM, IMP, and OXA-48-like) were primarily determined using a rapid immunochromatographic assay (NG-Test^®^ CARBA-5) and confirmed or further investigated by multiplex PCR for specific genes, as detailed in the Section 4. The distribution of carbapenemase genes identified among the CRPA and CRE isolates is presented below. Among the 65 CRPA isolates, 18 (27.7%) harbored carbapenemase genes. NDM was the most prevalent (14/18, 77.8%), followed by VIM (2/18, 11.1%) and IMP (2/18, 11.1%) (Table 4). No KPC or OXA-48-like producers were identified among CRPA isolates. Of the 55 CRE isolates, 33 (60.0%) were carbapenemase-producing. The most common enzyme was KPC (28/33, 84.8%), followed by NDM (4/33, 12.1%) and OXA-48-like (1/33, 3.0%). No CRE isolates produced VIM or IMP. Among KPC producers, *K. pneumoniae* accounted for the majority (25/28, 89.3%). The remaining 22 (40.0%) CRE isolates were non-carbapenemase producers, including *K. pneumoniae* (7), *E. coli* (7), *E. cloacae* (5), and *S. marcescens* (3).

The distribution of carbapenemase types among CRPA and CRE isolates is summarized in Table 4. To further investigate isolates that tested negative by NG-Test CARBA-5, multiplex PCR was performed on 26 isolates with elevated C/T minimum inhibitory concentrations (MICs), confirming the absence of KPC, NDM, VIM, IMP, and OXA-48-like genes. Additionally, all 69 NG-Test-negative isolates underwent modified carbapenem inactivation method (mCIM) testing. Of these, 67 yielded negative results, while two *E. cloacae* complex isolates produced inconclusive results. These findings suggest that no additional carbapenemase activity beyond the five major types was detected.

### 2.3. Antimicrobial Susceptibility Profiles

Susceptibility profiles varied significantly among non-CP CRPA, non-CP CRE, and KPC-producing CRE isolates (Table 5). Among non-CP CRPA isolates, susceptibility was highest to C/T (83.0%), followed by CZA (70.2%), MEV (66.0%), and IMR (63.8%). Notably, 17.0% and 19.1% of isolates exhibited intermediate or resistant profiles to IMR, respectively. Non-CP CRE isolates demonstrated high susceptibility to CZA (100%), MEV (95.5%), and IMR (81.8%). However, only 18.2% were susceptible to C/T, with both MIC_50_ and MIC_90_ values at 16 µg/mL, indicating considerable resistance (68.2%). KPC-producing CRE isolates retained high susceptibility to CZA (92.9%), MEV (96.4%), and IMR (82.1%). MEV exhibited the lowest MIC_50_ (0.03 µg/mL), suggesting excellent in vitro efficacy. A temporal comparison between pre-COVID-19 (2017–2019) and post-COVID-19 (2020–2022) isolates revealed no significant differences in susceptibility patterns (Supplementary Table S1).

## 3. Discussion

The carbapenemases produced by CRPA and CRE isolates were analyzed both clinically and microbiologically. The detection and characterization of these enzymes have significant implications for clinical practice and infection control [5,8]. Understanding carbapenemase profiles facilitates the selection of appropriate antimicrobial treatments, reduces therapeutic failure rates, and helps mitigate the spread of multidrug-resistant pathogens. Given these critical implications, continued surveillance of antimicrobial resistance patterns within healthcare settings is therefore essential. Identifying carbapenemases also assists healthcare institutions in developing targeted infection prevention strategies, minimizing the potential for nosocomial outbreaks.

CRE-infected patients were more likely to have underlying hematologic malignancies, receive immunosuppressive therapy, and experience significantly higher in-hospital mortality (63.6% vs. 18.5%, *p* < 0.001), underscoring the clinical severity of CRE bacteremia. Regarding primary sources of infection, primary bacteremia or neutropenic fever predominated among CRE patients, whereas urinary tract infections were the most frequent source among CRPA patients. Similar findings have been reported in previous Korean studies, although pneumonia and catheter-related infections were also commonly noted in CRE cases [15,16,17]. These differences may reflect the unique patient populations and medical practices at specialized institutions that primarily manage hematologic malignancies and cancer.

The high in-hospital mortality observed in CRE-infected patients in our study (63.6% vs. 18.5% for CRPA, *p* < 0.001), as noted earlier, warrants further discussion. Previous reports indicate that mortality rates for CRE infections typically range from 26% to 44%, and Korean studies specifically reported 14-day and 30-day mortality rates of 34.0% and 42.2%, respectively [17,18]. Additionally, the limited availability and delayed reimbursement approval for newer antibacterial agents in Korea—such as C/T, approved in 2017 but reimbursed only from 2022, and CZA, approved in 2022 and reimbursed in 2023—may also contribute to increased mortality. Further investigations into these factors are necessary to comprehensively understand and address this elevated mortality risk. The higher frequency of colistin use observed among patients infected with carbapenemase-producing isolates (both CP CRPA and CP CRE) likely reflects the limited therapeutic options available for these infections during the study period, prior to the widespread availability and reimbursement of novel agents in Korea. This underscores the significant treatment challenges posed by CP organisms, primarily due to limited therapeutic options resulting from extensive antimicrobial resistance and the variable efficacy of older last-resort agents.

The challenges in managing CRE and CRPA infections observed during our study period (2017–2022), which significantly overlapped with the COVID-19 pandemic, should also be considered in the broader context of pandemic-related impacts on antimicrobial resistance and usage. Although our study did not directly assess the specific impact of COVID-19 on the susceptibility patterns of the novel agents, it is noteworthy that national data from Korea (Kor-GLASS) suggest that overall antibiotic consumption, especially of broad-spectrum agents like cephalosporins, increased in the post-2021 period. This was accompanied by sustained or increased resistance rates in common Gram-negative pathogens to various antibiotics up to 2023 [12]. These national trends, including a slight increase in carbapenem consumption post 2021, suggest that the challenges of antimicrobial resistance and high antibiotic usage have persisted, and possibly intensified, in the healthcare landscape shaped by the pandemic. Factors such as disruptions to infection prevention and control measures, increased hospitalizations, and the complexity of managing critically ill patients may have contributed to these observations, underscoring the continued need for robust antimicrobial stewardship and surveillance.

Novel therapeutic agents, including C/T, CZA, IMR, and MEV, have been developed to address carbapenem resistance. However, their effectiveness varies according to the specific carbapenemase type. For example, while CZA is active against KPC and OXA-48-like carbapenemases, it lacks activity against metallo-β-lactamases (MBLs) such as NDM and VIM [19]. IMR demonstrates activity against KPC producers but is not effective against MBLs or OXA-48-like enzymes [13]. C/T is generally ineffective against most carbapenemases, including KPC and MBLs [19]. MEV is effective against KPC-producing isolates but not against MBLs or most OXA-type carbapenemases [13]. Consequently, precise identification of carbapenemase genes is critical for determining the most appropriate antimicrobial treatment. Previous global studies have reported varying prevalence rates of carbapenemase-producing CRPA: 32% in China, 57% in Australia and Singapore, and 2% in the USA [10,20]. In our study, 27.7% of CRPA isolates produced carbapenemases, slightly lower than previously reported rates in the Asia–Pacific region. Meanwhile, over 50% of CRE isolates produced carbapenemases, predominantly KPC enzymes, followed by NDM and OXA-48-like enzymes. Global reports indicate CP-CRE prevalence rates ranging from 20.7% in Japan to 85.7% in China, with Korean data indicating intermediate prevalence (approximately 42.9–58.1%) [21,22,23,24,25,26].

In this study, we employed the mCIM to detect possible carbapenemase activities in negative isolates using NG-test CARBA-5 and multiplex PCR assays. However, no additional carbapenemase activity beyond the five major enzymes (KPC, NDM, VIM, IMP, and OXA-48-like) was identified. Only two isolates showed consistently inconclusive results upon repeated mCIM testing. Given the high concordance among NG-test CARBA-5, multiplex PCR, and mCIM, our findings suggest that mCIM may not be routinely necessary in Korean settings when both immunochromatographic and molecular assays yield negative results. Generally, the mCIM is recognized for its broad ability to detect a variety of carbapenemases, including Ambler class A, B, and D enzymes, as demonstrated in comprehensive evaluations in previous studies [27,28]. In our study, among the 69 isolates that tested negative for the five major carbapenemases by NG-test CARBA-5 and multiplex PCR, no isolates were definitively positive by mCIM. This finding, in the context of the known broad detection capabilities of mCIM, might reflect the local epidemiology, where carbapenemases other than five major types are infrequent, as supported by Korean surveillance data [24,25]. Nevertheless, the absence of more comprehensive genotypic testing, such as NGS, in these five major negative isolates is a limitation in definitively assessing the prevalence of other carbapenemases and their mCIM reactivity in our cohort. Further studies with larger isolate collections and diverse clinical sources are necessary to validate these conclusions comprehensively.

Data on the susceptibility of CRPA and CRE to novel β-lactam/β-lactamase inhibitors remain limited in Korea. Previous Korean studies reported higher susceptibility rates of non-carbapenemase-producing CRPA to C/T (95.2%) and CZA (71.4%) in isolates collected during 2006–2007 [14]. Another recent study using lower respiratory tract isolates demonstrated high susceptibility rates to C/T (90.1%) and CZA (97.1%) in *Enterobacterales* and *P. aeruginosa*, respectively [29]. However, these earlier studies included very few carbapenemase-producing isolates, limiting their relevance to the current epidemiologic context. In contrast, our findings, based on more recent isolates, showed comparatively lower susceptibility rates among non-CP CRPA to C/T (83.0%) and CZA (70.2%), potentially reflecting increased resistance due to broader clinical use of these antibiotics.

Our study indicated high susceptibility rates for non-CP CRE isolates to CZA (100%), MEV (95.5%), and IMR (81.8%), whereas susceptibility to C/T was notably lower (18.2%). For KPC-producing CRE isolates, CZA, IMR, and MEV demonstrated high susceptibility (92.9%, 82.1%, and 96.4%, respectively), supporting their clinical efficacy against these resistant organisms. Considering MIC values, CZA, IMR, and MEV should be preferred empirically over C/T in regions with high carbapenemase prevalence, particularly KPC producers.

The findings of this study offer several implications for antimicrobial stewardship guidelines in Korean hospitals. Firstly, the contemporary local susceptibility data for novel agents against CRE and CRPA, particularly the high activity of CZA, IMR, and MEV against KPC-producing CRE, can help refine empirical therapy guidelines for severe infections. Secondly, the observed distribution of carbapenemase types, with a predominance of KPC and NDM, underscores the importance of rapid and accurate carbapenemase genotyping to guide optimal drug selection, as the efficacy of these novel agents varies significantly by enzyme type. For instance, our data support the preferential use of CZA, IMR, or MEV for KPC producers, while highlighting the limited utility of C/T against most CP-CRE. These insights can aid in developing more targeted and effective antimicrobial stewardship strategies, promoting the judicious use of new antibiotics and preserving their efficacy.

This study has certain limitations. Firstly, we only screened for five major carbapenemase genes (KPC, NDM, VIM, IMP, and OXA-48-like), and did not perform more comprehensive genotypic analyses, such as next-generation sequencing (NGS). This approach might have missed other, less common carbapenemase genes (e.g., GES, IMI) or other resistance mechanisms that could be present in our isolates, including those that tested negative by mCIM. Secondly, isolates were derived exclusively from bloodstream infections; future research incorporating isolates from diverse clinical specimens (e.g., urine, respiratory samples) is warranted. Thirdly, this was a single-center study, and the findings may not be generalizable to other institutions with different patient populations or antimicrobial prescribing practices. Finally, this study did not perform specific tests (e.g., population analysis profiles) to systematically investigate heteroresistance. Standard susceptibility testing methods, such as those used in our study, may not always detect low-frequency resistant subpopulations, which can be a clinically relevant phenomenon and a potential contributor to treatment failure with newer β-lactam/β-lactamase inhibitors in CRE infections, as highlighted in the recent literature [30]. Larger, multi-center studies incorporating comprehensive genotypic characterization and heteroresistance testing are needed to clarify these potential relationships and further delineate the resistance landscape.

## 4. Materials and Methods

### 4.1. Study Population and Study Design

This retrospective study was conducted at Pusan National University Hospital, a 1300-bed tertiary care teaching hospital with eight intensive care units, located in Busan, Republic of Korea. Blood isolates of CRE and CRPA were collected from patients with documented bacteremia between September 2017 and September 2022. Notably, the study period preceded the routine clinical use of novel β-lactam/β-lactamase inhibitor combinations (C/T, CZA, IMR, and MEV) at the study institution.

Clinical data were obtained through retrospective review of electronic medical records. Collected variables included demographic characteristics (age and sex), underlying comorbidities, and previous colonization or infection with MDROs, including methicillin-resistant *Staphylococcus aureus* (MRSA), multidrug-resistant *Acinetobacter baumannii* (MRAB), CRPA, vancomycin-resistant *Enterococcus* (VRE), and ESBL-producing organisms. Information on the use of medical devices at the time of bacteremia (central venous catheter, mechanical ventilation, indwelling urinary catheter, or nasogastric tube), antimicrobial treatments administered, and in-hospital mortality was also collected. The primary source of bacteremia was determined by infectious disease specialists through comprehensive chart reviews.

### 4.2. Bacterial Isolates

Blood isolates identified as intermediate or resistant to carbapenems (ertapenem, imipenem, or meropenem) according to the CLSI breakpoints [31] by the Vitek^®^2 automated identification system (bioMérieux, Marcy-l’Étoile, France) were classified as CRE or CRPA and included in the study. For patients with multiple isolates, only the first isolate was included if subsequent isolates were collected within one month. If the interval between isolates exceeded one month, each isolate was analyzed independently. All isolates were preserved in skim milk at −80 °C until further experimentation. A total of 120 non-duplicate bloodstream isolates (65 CRPA and 55 CRE) meeting these criteria were collected during the study period (September 2017–September 2022). As this was a descriptive epidemiological study based on retrospectively collected isolates over a defined period, a formal a priori sample size calculation was not performed.

### 4.3. Carbapenemase Detection

Carbapenemase production was screened using the NG-Test^®^ CARBA-5 kit (NG Biotech, Guipry, France), a rapid immunochromatographic assay capable of detecting the five major carbapenemases: KPC, NDM, VIM, IMP, and OXA-48-like enzymes. Briefly, bacterial suspensions were prepared by mixing three colonies in 150 µL extraction buffer, and 100 µL of the suspension was transferred to the test cassette. Results were interpreted visually after 15 min. To confirm the absence of carbapenemase genes, multiplex polymerase chain reaction (PCR) was performed on isolates that tested negative by the NG-Test. Specifically, multiplex PCR testing targeted isolates with C/T MICs ≥ 2 µg/mL (CRPA isolates) or ≥1.5 µg/mL (CRE isolates), as determined by the gradient diffusion method. Primers and PCR cycling conditions were adapted from previously validated protocols [32]. Additionally, the mCIM was conducted on negative isolates using both NG-test CARBA-5 and multiplex PCR. The mCIM tests were performed according to Clinical and Laboratory Standards Institute (CLSI) guidelines [31]. Interpretations of the mCIM results followed CLSI criteria: isolates producing zones of inhibition ≤ 15 mm or those with pinpoint colonies within a 16–18 mm zone were classified as carbapenemase-positive, isolates showing clear zones ≥ 19 mm were classified as negative, and ambiguous results (zones 16–18 mm without pinpoint colonies or ≥19 mm with pinpoint colonies) were classified as inconclusive.

### 4.4. Antimicrobial Susceptibility Testing

Antimicrobial susceptibility testing for the four novel β-lactam/β-lactamase inhibitors (C/T, CZA, IMR, and MEV) was performed on a selection of these isolates based on their carbapenemase status. C/T susceptibility testing was limited to non-carbapenemase-producing (non-CP) isolates (47 CRPA and 22 CRE), as C/T exhibits limited activity against most carbapenemase-producing organisms, particularly KPC and metallo-β-lactamase (MBL) producers. Consequently, KPC-producing CRE (*n* = 28) were excluded from C/T testing. For CZA, IMR, and MEV, testing was performed on non-CP CRPA (*n* = 47), non-CP CRE (*n* = 22), and KPC-producing CRE (*n* = 28) isolates, resulting in a total of 97 unique isolates being tested for these three agents (Figure 1). The remaining 23 isolates, comprising carbapenemase-producing CRPA (*n* = 18, predominantly MBL producers) and CRE isolates harboring MBLs or OXA-48-like enzymes (*n* = 5), were not subjected to susceptibility testing for these four novel agents due to their known resistance profiles or the specific focus of the current investigation on non-MBL and KPC-producing isolates for these newer agents. Susceptibility to C/T, CZA, IMR and MEV for the selected isolates was then assessed primarily using the broth microdilution method, following the CLSI guidelines [33]. 

Broth microdilution was performed using commercially available Sensititre™ Gram-negative MDRGNXXF MIC plates (Thermo Fisher Scientific, Waltham, MA, USA). Briefly, bacterial inocula were prepared by suspending 3–5 bacterial colonies in sterile demineralized water and adjusting turbidity to a 0.5 McFarland standard. Then, 10 µL of the adjusted bacterial suspension was inoculated into each well containing serial dilutions of antimicrobial agents. Plates were sealed and incubated for 18–24 h at 35 °C under ambient conditions. The MIC was defined as the lowest antimicrobial concentration that completely inhibited visible bacterial growth.

In addition to broth microdilution, a gradient diffusion method for C/T were used for selected isolates. Specifically, Etest (bioMérieux, Marcy-l’Étoile, France) was employed. Bacterial suspensions were prepared from overnight cultures and adjusted to a 0.5 McFarland standard using sterile saline. Using a sterile cotton swab, the inoculum was evenly spread over the surface of Mueller-Hinton II agar plates (Becton Dickinson, Sparks, MD, USA). Once the surface was fully dry, antimicrobial strips were placed on the agar and plates were incubated inverted at 34–36 °C for 16–20 h. The testable MIC range was 0.016/4–256/4 µg/mL.

Susceptibility breakpoints for C/T, CZA, IMR, and MEV were interpreted according to CLSI M100 ED34:2024 and the European Committee on Antimicrobial Susceptibility Testing (EUCAST) version 14.0 (Supplementary Tables S2 and S3, available in the Supplementary Materials) [31,34].

### 4.5. Data Analysis and Ethics

Statistical analyses were performed using MedCalc^®^ software, version 22.021 (MedCalc Software Ltd., Ostend, Belgium), IBM SPSS Statistics (version 27, IBM Corp., Armonk, NY, USA), and R (version 4.4.1; R Foundation for Statistical Computing, Vienna, Austria) and RStudio (version 2024.09.0; RStudio, Boston, MA, USA). Continuous variables are presented as means and standard deviations, and categorical variables as frequencies and percentages. Comparisons between categorical variables were performed using Pearson’s χ^2^ test. Fisher’s exact test was used when the expected frequency in any cell was less than 5 or when an observed cell frequency was zero. Continuous variables were analyzed using the *t*-test. A *p* value < 0.05 was considered statistically significant.

We also performed subgroup analyses comparing antimicrobial susceptibility between isolates collected during pre-COVID-19 (2017–2019) and post-COVID-19 (2020–2022) periods. This study was approved by the Institutional Review Board (IRB) of Pusan National University Hospital (approval number: 2212-001-121). The requirement for informed consent was waived due to the retrospective observational nature of the study. All study procedures were conducted in accordance with the guidelines of the Declaration of Helsinki. A portion of this study was previously presented as a conference abstract at IDWeek 2023; however, the current manuscript includes expanded datasets, updated analyses, and additional clinical correlations [35].

## 5. Conclusions

Our study provides essential updated data on the prevalence, clinical characteristics, and antimicrobial susceptibility patterns of CRE and CRPA isolates in Korea. The selection of appropriate empirical antibiotics based on detailed susceptibility profiles and carbapenemase screening can significantly improve patient outcomes and guide effective infection control strategies.

## Figures and Tables

**Table 1:** Baseline characteristics and comorbidities of patients with bloodstream infections caused by CRPA and CRE.

Characteristics	CRPA (*n* = 65)	CRE (*n* = 55)	*p *^a^
Age, years	65 (37–88) ^b^	55 (21–88) ^b^	0.008 ^c^
Sex, male	39 (60.0)	37 (67.3)	0.451
Comorbidities			
Cardiovascular disease	3 (4.6)	5 (9.1)	0.467
Hypertension	19 (29.2)	16 (29.1)	1.000
Heart failure	5 (7.7)	10 (18.2)	0.101
Chronic lung disease	0 (0.0)	4 (7.3)	0.041
Cerebrovascular accident	12 (18.5)	4 (7.3)	0.105
Dementia	1 (1.5)	1 (1.8)	1.000
Diabetes	19 (29.2)	15 (27.3)	0.842
Liver disease	10 (15.4)	9 (16.4)	1.000
Chronic kidney disease	9 (13.8)	6 (10.9)	0.783
Solid tumor	25 (38.5)	26 (47.3)	0.359
Hematologic malignancy	3 (4.6)	14 (25.5)	<0.001
Immunosuppressive treatment	10 (15.4)	33 (60.0)	<0.001
HIV/AIDS	1 (1.5)	0 (0.0)	1.000

^a^ Chi-squared test or Fisher’s exact test, as appropriate, unless stated otherwise; ^b^ median (range); ^c^ independent samples *t*-test. Numbers within parentheses are percentages unless stated otherwise. Abbreviations used are defined in the list at the end of the manuscript.

**Table 2:** Primary origin of bacteremia and devices present at onset of infection in patients with bloodstream infections caused by CRPA and CRE.

Characteristics	CRPA (*n* = 65)	CRE (*n* = 55)	*p *^a^
Primary origin of bacteremia			
Intraabdominal infection	25 (38.5)	18 (32.7)	0.569
Urinary tract infection	16 (24.6)	1 (1.8)	<0.001
Catheter-related infection	10 (15.4)	13 (23.6)	0.352
Pneumonia	5 (7.7)	8 (14.5)	0.253
Primary bacteremia or neutropenic fever	4 (6.2)	13 (23.6)	0.008
Soft tissue, musculoskeletal infection	3 (4.6)	2 (3.6)	1.000
Others	2 (3.1)	0 (0.0)	0.499
Devices present at onset of infection			
Central venous catheter	23 (35.4)	39 (70.9)	<0.001
Mechanical ventilator	4 (6.2)	16 (29.1)	0.001
Indwelling urinary catheter	25 (38.5)	27 (49.1)	0.271
Nasogastric tube	13 (20.0)	21 (38.2)	0.041

^a^ Chi-squared test or Fisher’s exact test, as appropriate, unless stated otherwise. Numbers within parentheses are percentages. Abbreviations used are defined in the list at the end of the manuscript.

**Table 3:** Previous colonization with MDROs, antimicrobial therapy for CRPA or CRE, and in-hospital mortality in patients with bloodstream infections caused by CRPA and CRE.

Characteristics	CRPA (*n* = 65)	CRE (*n* = 55)	*p *^a^
Previous colonization with MDROs			
MRSA	9 (13.8)	9 (16.4)	0.799
MRAB	10 (15.4)	6 (10.9)	0.593
CRPA	25 (38.5)	6 (10.9)	<0.001
CRE	15 (23.1)	23 (41.8)	0.032
VRE	7 (10.8)	11 (20.0)	0.202
ESBL-producing bacteria	16 (24.6)	15 (27.3)	0.835
Antimicrobial therapy for CRPA or CRE			
Colistin alone	18 (27.7)	11 (20.0)	0.394
Colistin + carbapenem	1 (1.5)	10 (18.2)	0.003
Cephalosporin alone	17 (26.2)	6 (10.9)	0.039
Others	5 (7.7)	5 (9.1)	1.000
Tigecycline	0 (0.0)	2 (3.6)	0.208
None for CRPA or CRE	20 (30.8)	21 (38.2)	0.713
In-hospital mortality	12 (18.5)	36 (63.6)	<0.001

^a^ Chi-squared test or Fisher’s exact test, as appropriate, unless stated otherwise. Numbers within parentheses are percentages. Abbreviations used are defined in the list at the end of the manuscript.

**Table 4:** Distribution of carbapenemase-producing and non-producing isolates among CRPA and CRE.

Carbapenemase Types and Species	No. (%)
**CRPA (*n* = 65)**	
**Non-CP CRPA**	47 (72.3)
**CP CRPA**	18 (27.7)
NDM	14 (21.5)
VIM	2 (3.1)
IMP	2 (3.1)
OXA-48-like	0 (0.0)
KPC	0 (0.0)
**CRE (*n* = 55)**	
**Non-CP CRE**	22 (40.0)
*Klebsiella pneumoniae*	7 (12.7)
*Escherichia coli*	7 (12.7)
*Enterobacter cloacae*	5 (9.1)
*Serratia marcescens*	3 (5.5)
**CP CRE**	33 (60.0)
KPC	
*Klebsiella pneumoniae*	25 (45.5)
*Klebsiella oxytoca*	1 (1.8)
*Escherichia coli*	1 (1.8)
*Serratia marcescens*	1 (1.8)
NDM	
*Enterobacter cloacae*	3 (5.5)
*Escherichia coli*	1 (1.8)
OXA-48-like	
*Escherichia coli*	1 (1.8)

Abbreviations used are defined in the list at the end of the manuscript.

**Table 5:** MIC distributions and in vitro susceptibility of non-carbapenemase-producing CRPA, non-carbapenemase-producing CRE, and KPC-producing CRE isolates to novel β-lactam/β-lactamase inhibitor combinations.

Isolate Type	Antimicrobial Agent	MIC Range (µg/mL)	MIC50 (µg/mL)	MIC90 (µg/mL)	Susceptible (%)	Intermediate (%)	Resistant (%)
**Non-CP CRPA** **(*n* = 47)**	C/T	0.25–>8	1	>8	83.0	6.4	10.6
	CZA	0.25–>32	4	32	70.2	–	29.8
	IMR	0.25–>16	2	16	63.8	17.0	19.1
	MEV	0.25–>16	8	>16	66.0	–	34.0
**Non-CP CRE** **(*n* = 22)**	C/T	0.5–>8	>8	>8	18.2	13.6	68.2
	CZA	0.25–8	1	4	100	–	0
	IMR	≤0.06–2	0.25	2	81.8	18.2	0
	MEV	≤0.03–16	0.25	2	95.5	–	4.5
**KPC-producing CRE** **(*n* = 28)**	CZA	0.25–>32	1	4	92.9	–	7.1
	IMR	0.125–16	0.125	2	82.1	10.7	7.1
	MEV	≤0.015–8	0.03	4	96.4	3.6	0

Abbreviations used are defined in the list at the end of the manuscript.

## Data Availability

The datasets generated and analyzed during the current study are available from the corresponding author upon reasonable request.

## Supplementary Materials

The following supporting information can be downloaded at: https://www.mdpi.com/article/10.3390/antibiotics14070649/s1, Table S1: Antimicrobial susceptibility of CRE and CRPA isolates to novel agents in the pre- and post-COVID-19 periods; Table S2: Clinical breakpoints for β-lactam/β-lactamase inhibitor combinations against *Enterobacterales*; Table S3: Clinical breakpoints for β-lactam/β-lactamase inhibitor combinations against *Pseudomonas aeruginosa*.

## Abbreviations

The following abbreviations are used in this manuscript:

AmpC	AmpC β-lactamases
AST	Antimicrobial susceptibility testing
BMD	Broth microdilution
C/T	Ceftolozane/tazobactam
CZA	Ceftazidime/avibactam
CP	Carbapenemase-producing
COPD	Chronic obstructive pulmonary disease
CRE	Carbapenem-resistant *Enterobacterales*
CROs	Carbapenem-resistant organisms
CRPA	Carbapenem-resistant *Pseudomonas aeruginosa*
CLSI	Clinical and Laboratory Standards Institute
ESBL	Extended-spectrum β-lactamase
EUCAST	European Committee on Antimicrobial Susceptibility Testing
GES	Guiana-extended-spectrum
HIV/AIDS	Human immunodeficiency virus/acquired immunodeficiency syndrome
IMP	Imipenem-hydrolysing β-lactamase
IMR	Imipenem/cilastatin/relebactam
IRB	Institutional Review Board
KPC	*Klebsiella pneumoniae* carbapenemase
mCIM	Modified carbapenem inactivation method
MBLs	Metallo-β-lactamases
MDR	Multidrug-resistant
MDROs	Multidrug-resistant organisms
MEV	Meropenem/vaborbactam
MIC	Minimum inhibitory concentration
MRAB	Multidrug-resistant *Acinetobacter baumannii*
MRSA	Methicillin-resistant *Staphylococcus aureus*
NDM	New Delhi metallo-β-lactamase
NGS	Next-generation sequencing
Non-CP	Non-carbapenemase-producing
OXA	Oxacillinase
PCR	Polymerase chain reaction
VIM	Verona integron-encoded metallo-β-lactamase
VRE	Vancomycin-resistant *Enterococcus*
