# Supplementary material for: In Vitro Activity of Novel β-Lactam/β-Lactamase Inhibitors Against Carbapenem-Resistant Pseudomonas aeruginosa and Enterobacterales in Korea
Source: Antibiotics (Basel). 2025 Jun 26;14(7):649. doi: 10.3390/antibiotics14070649 (PMC12291907; doi:10.3390/antibiotics14070649)
Supplement: Supplementary file 1 [file antibiotics-14-00649-s001.zip › antibiotics-3670858-supplementary.pdf]

Supplementary Tables

Supplementary Table S1. Antimicrobial susceptibility of CRE and CRPA isolates to novel agents in the pre- and post-COVID-19 periods

Susceptible (%)	CRE (N = 50)			CRPA (N = 47)		
	Pre (n = 21)	Post (n = 29)	*P*	Pre (n = 18)	Post (n = 29)	*P*
C/T	3 (14.3)	1 (3.4)	0.297	16 (88.9)	23 (79.3)	0.692
CZA	20 (95.2)	28 (96.6)	1.000	15 (83.3)	18 (62.1)	0.191
IMR	17 (81.0)	24 (82.8)	1.000	13 (72.2)	17 (58.6)	0.533
MEV	20 (95.2)	28 (96.6)	1.000	12 (66.7)	19 (65.5)	1.000

Abbreviations: CRE, carbapenem-resistant *Enterobacterales*; CRPA, carbapenem-resistant *Pseudomonas aeruginosa*; COVID-19, coronavirus disease-19; C/T, ceftolozane/tazobactam; CZA, ceftazidime/avibactam; IMR, imipenem/relebactam; MEV, meropenem/vaborbactam

Supplementary Table S2. Clinical breakpoints for β-lactam/β-lactamase inhibitor combinations against *Enterobacterales*

Antibiotics	Susceptible (mg/L)	Intermediate (mg/L)	Resistant (mg/L)	Breakpoint Reference
Ceftolozane/tazobactam	≤2/4	4/4	≥8/4	CLSI M100 ED34:2024
Ceftazidime/avibactam	≤8/4	Not defined	≥16/4	CLSI M100 ED34:2024
Meropenem/vaborbactam	≤4/8	8/8	≥16/8	CLSI M100 ED34:2024
Imipenem/relebactam	≤1/4	2/4	≥4/4	CLSI M100 ED34:2024

Note: Breakpoints are expressed as drug/inhibitor (mg/L). "Not defined" indicates no intermediate category according to reference.

Supplementary Table S3. Clinical breakpoints for β-lactam/β-lactamase inhibitor combinations against *Pseudomonas aeruginosa*

Antibiotics	Susceptible (mg/L)	Intermediate (mg/L)	Resistant (mg/L)	Breakpoint Reference
Ceftolozane/tazobactam	≤4/4	8/4	≥16/4	CLSI M100 ED34:2024
Ceftazidime/avibactam	≤8/4	Not defined	≥16/4	CLSI M100 ED34:2024
Meropenem/vaborbactam	≤8/8	Not defined	≥16/8	EUCAST Version 14.0 (2024)
Imipenem/relebactam	≤2/4	4/4	≥8/4	CLSI M100 ED34:2024

Note: Breakpoints are expressed as drug/inhibitor (mg/L). "Not defined" indicates no intermediate category according to reference.
